# Evaluation of Static Balance in Children with Cerebral Palsy Using an Innovative Image Processing Software

**DOI:** 10.3390/healthcare13212682

**Published:** 2025-10-23

**Authors:** Zekiye Basaran, Halil Ibrahim Celik, Onder Polat, Bulent Elbasan

**Affiliations:** 1 Department of Physiotherapy and Rehabilitation, Gazi University, 06560 Ankara, Turkey; bulentelbasan@gazi.edu.tr; 2 Bilge Child Special Education and Rehabilitation Center, 06800 Ankara, Turkey; fizyoterapist70@gmail.com; 3 Department of Electrical and Electronics Engineering, Gaziantep Islamic Science and Technology University, 27010 Gaziantep, Turkey; onder.polat@gibtu.edu.tr

**Keywords:** cerebral palsy, static balance, image processing, validity, postural control, pediatric rehabilitation

## Abstract

**Background:** Impaired balance is one of the most common and functionally limiting problems in children with cerebral palsy (CP), significantly affecting their motor abilities and quality of life. Although force platforms are considered the gold standard for evaluating postural stability, they are often costly, non-portable, and require specialized laboratory environments, limiting their accessibility in routine clinical settings. **Objective:** This study aimed to develop a novel software program based on image processing techniques to assess static balance in children with CP and to evaluate its validity against traditional force platform measurements. **Methods:** A total of 83 children aged 5–15 years (63 with CP, GMFCS levels I–II; 20 healthy controls) participated. Static balance was assessed under four different standing conditions using both a force platform and a newly developed video-based software tool. The software utilized the frame difference method to detect center of mass movements, and parameters such as velocity and total displacement were calculated. Correlation analyses were conducted between the image processing and force platform data. **Results:** The software demonstrated moderate to strong positive correlations with force platform parameters in the majority of test conditions, particularly when participants stood with eyes open. In more challenging balance scenarios (e.g., eyes closed, feet together), correlations were weaker but still significant. **Conclusions:** The findings suggest that this image-based software is a valid, low-cost, and portable alternative for static balance assessment in children with CP. It has the potential for use in diverse clinical or home settings, supporting individualized rehabilitation strategies.

## 1. Introduction

Cerebral palsy (CP) is a group of permanent disorders of movement and posture development caused by non-progressive lesions in the immature brain. These impairments often lead to limitations in motor function, activity, and participation [1,2]. One of the most common and functionally disabling issues in children with CP is impaired balance, which results from a combination of abnormal muscle tone, poor postural control, deficits in motor planning, and sensory integration dysfunctions [3]. Balance impairments negatively affect not only the child’s ability to maintain a stable posture but also their functional independence and quality of life [4].

In individuals with CP, balance plays a critical role in performing basic motor tasks such as sitting, standing, and walking. Impaired balance increases the risk of falls and limits the child’s participation in daily life and social activities [5]. Therefore, accurate assessment of balance is essential for determining appropriate rehabilitation goals and tracking clinical progress. While both dynamic and static balance are important for postural stability, static balance is often a prerequisite for more complex movements and is particularly relevant in children with severe motor impairments [6,7].

Traditional clinical balance assessment tools, such as observational scales or simple functional tests, may lack the sensitivity and objectivity needed to detect subtle postural instabilities, especially in pediatric neurological populations [8]. Furthermore, many of these tools require trained evaluators, are time-consuming, or lack standardization. With the growing emphasis on evidence-based practice in rehabilitation, there is a need for more objective, reliable, and accessible methods for balance evaluation in children with CP [9].

Recent advances in technology have enabled the integration of image-processing and computer vision techniques into clinical assessment protocols [10]. Among these, video-based motion analysis systems offer a non-invasive, cost-effective, and portable alternative for evaluating postural control and movement patterns [11]. Video-based image processing techniques can capture and quantify body sway, center of pressure deviations, and other kinematic parameters that reflect balance performance without the need for complex instrumentation or specialized laboratory environments [12].

Video-based image processing techniques fundamentally operate by analyzing changes between consecutive video frames. Commonly employed approaches include frame difference, background subtraction, optical flow, and keypoint/pose estimation [13]. Among these, the frame difference technique isolates moving regions by calculating pixel-level differences between two successive frames under static camera conditions. This method is computationally lightweight and fast, making it suitable even for low-cost devices with limited processing capacity. In contrast, deep learning–based pose estimation methods (e.g., OpenPose, MediaPipe, DeepLabCut) provide more detailed, joint-level information but require higher computational resources and, in some cases, additional calibration [14,15]. Frame-difference based approaches are widely reported in the literature as efficient and feasible solutions for real-world applications [15,16].

The applicability of these techniques is of particular interest in clinical practice, especially within pediatric neurological populations [12,17]. The application of video-based image processing in the evaluation of static balance is particularly promising for children with CP, who may have difficulty complying with traditional instrumented assessments [18]. By providing objective and quantitative data, such systems can enhance clinical decision-making and facilitate personalized rehabilitation planning. Moreover, the scalability and adaptability of video-based systems support their potential for broader implementation in both clinical and home-based settings.

In recent years, out-of-laboratory and markerless video-based motion analysis solutions have advanced rapidly. In particular, two-dimensional (2D) markerless approaches enable the extraction of joint keypoints and the automated estimation of spatio-temporal kinematic parameters using either a single two-dimensional color image or a combination of two-dimensional color images and depth cameras [19]. These approaches have been applied to monitor movement disorders and to automatically classify functional movements in neuromotor conditions such as cerebral palsy (CP), and have been proposed as practical and portable alternatives [17]. However, these methods present certain limitations, including depth and three-dimensional (3D) ambiguities, errors due to occlusion or clothing, and the computational cost of some deep learning–based solutions.

In the present study, in order to overcome the limitations of more complex markerless systems, we developed a software solution based on a single camera and frame-difference algorithms with low computational cost. This provides a practical and accessible method for static balance assessment that can be readily applied in both clinical and home environments. Furthermore, the outputs of the software were directly compared with force platform center of pressure (COP) data to evaluate its validity. In this respect, the study differentiates itself from the majority of systems in the literature, which primarily focus on gait analysis and require high-end hardware, by offering a more feasible alternative for clinical practice.

The remainder of the manuscript is structured as follows: Section 2 (Methods) describes the participants, experimental setup, video acquisition, core components of the image-processing software, and data acquisition and preprocessing steps in detail. Section 3 (Results) presents the statistical comparisons and modeling findings between the software outputs and force platform results. Section 4 (Discussion) provides a comparison of the findings with the existing literature, a discussion of the strengths and limitations of the software, and recommendations for future development. Finally, Section 5 (Conclusions) summarizes the study and offers suggestions for clinical and research applications.

## 2. Materials and Methods

### 2.1. Participants

The study included individuals aged 5–15 years diagnosed with CP who applied to the Pediatric Rehabilitation Unit of the Department of Physiotherapy and Rehabilitation. After informing the parents about the study’s purpose and procedures, written informed consent was obtained from those who agreed to participate using a “Voluntary Informed Consent Form.” A total of 90 children, including 70 with CP and 20 healthy children, were initially assessed. Seven participants from the CP group were excluded for various reasons (failure to meet inclusion criteria or incomplete data), resulting in a final sample of 83 children.

The CP group consisted of ambulatory children at Gross Motor Function Classification System (GMFCS) levels I–II, without severe visual, auditory, or cognitive impairments. The healthy control group included children without any neurological or orthopedic conditions. The inclusion criteria were: being between 5 and 15 years of age, ability to stand independently, and ability to maintain a static posture for at least 30 s. Exclusion criteria included a history of severe musculoskeletal injury, epileptic seizures, visual or auditory impairments, or comorbid severe chronic diseases.

Written informed consent was obtained from the parents or guardians of all participants. Furthermore, the requisite permissions were acquired from the Ethics Committee of Gazi University for the study (Approval No: E-77082166-302.08.01-344231).

### 2.2. Data Collection Tools

#### 2.2.1. Balance Assessment Protocol

A total of 83 children meeting the inclusion criteria participated in the study. Motor levels were assessed using the GMFCS. Balance assessments involved collecting Center of Pressure (COP) movement data and video recordings of children standing on a force plate under the following four conditions:

1. Condition: Standing with eyes open, feet shoulder-width apart, arms alongside the body (30 s).
2. Condition: Standing with eyes closed, feet shoulder-width apart, arms alongside the body (30 s).
3. Condition: Standing with eyes open, feet together, arms alongside the body (30 s).
4. Condition: Standing with eyes closed, feet together, arms alongside the body (30 s).

In all conditions, participants were instructed to stand still for 30 s with minimal postural sway, during which simultaneous video recordings were obtained. To ensure optimal motion tracking and to minimize clothing-related artifacts, children were assessed wearing minimal clothing (underwear only), in accordance with ethical and privacy standards. To standardize the recordings, all videos were captured in a controlled environment with a white background and floor, stable lighting and temperature, and the same camera mounted on a tripod. The clinical assessment for each child took approximately 15 min. Demographic characteristics, including gender, age, height, weight, affected area, and GMFCS level, were recorded.

#### 2.2.2. Center of Pressure (COP) Analysis

Assessment of static balance requires evaluating the movement of the center of mass (COM); however, direct measurement is complex and typically requires multisensor kinematic systems. Therefore, the center of pressure (COP), the projection of COM onto the support surface, is commonly used as an indirect measure [20]. COP reflects the weighted average of all pressures acting on the support surface and is influenced by the position and motion of the head, trunk, pelvis, and limbs [21].

In this study, COP data were collected using a Kistler force plate (Type 9260AA, 40 × 60 cm; Kistler Instruments AG, Winterthur, Switzerland). Children stood on the platform for a total of 2 min across four different 30-s trials. Raw COP data were recorded using MARS^®^ software (4.0 Full/Part Version, Kistler Instruments, Winterthur, Switzerland) and processed in MATLAB (Version 2018b, Natick, MA, USA). Analog signals were digitized at a sampling rate of 100 Hz and filtered using a fourth-order 10 Hz low-pass Butterworth filter to reduce noise. COP trajectories were analyzed in the x (mediolateral direction), y (anteroposterior direction), and r (radial resultant) directions [22]. For each axis, the mean velocity, standard deviation, and total displacement parameters were calculated (Table 1). These parameters reflect the temporal and spatial variability of the center of pressure, thereby enabling an objective assessment of postural control.

#### 2.2.3. Image Processing

Video recordings were obtained in MP4 format using a Huawei P40 lite JNY-LX1 smartphone camera. During the image processing stage, the videos were converted into AVI format to allow analysis within the MATLAB R2018b environment (MathWorks, Natick, MA, USA). The rationale for this conversion was that AVI format provides greater stability during the frame extraction process in MATLAB, reduces data loss, and enables noise-free processing.

The developed image processing software consisted of four main steps:

(a) Preprocessing: A region of interest covering each participant’s trunk was defined in the videos. This cropping procedure reduced background-related noise and ensured that information was extracted only from the relevant body region. Subsequently, the images were converted to grayscale, and normalization was applied to minimize brightness variations.

(b) Motion Detection: Pixel differences were calculated between consecutive frames within the ROI using the frame-difference method. For each pixel, the difference in values across consecutive frames was compared against a predefined threshold. Pixels with a difference below 0.15 were classified as 0 (static), whereas those above 0.15 were classified as 1 (moving). Thus, a binary motion image was generated for each frame [23].

(c) Noise Filtering: Isolated pixel errors and small regions in the binary images were eliminated using morphological operations. This step resulted in a more stable and distinct silhouette representing trunk movement.

(d) Feature Extraction: The geometric center (COM) of the silhouette was calculated for each frame. By tracking the movement of COM coordinates across frames, three primary parameters were derived in the mediolateral (x), anteroposterior (y), and radial (r) directions: mean velocity, standard deviation, and total displacement. These parameters extracted from the image processing software were structured in the same format as the force platform parameters, enabling direct comparison (Table 1). Pixel-based measurements were normalized using a scaling method for comparison with COP data.

**Figure 1:**
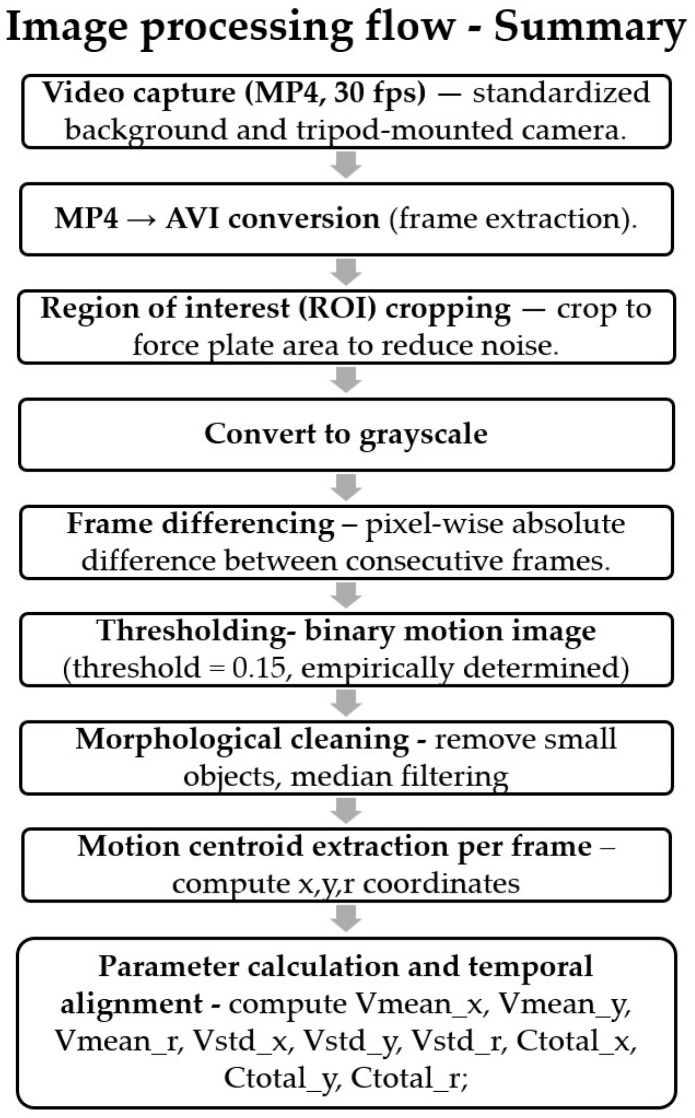
Steps implemented in image processing software.

The steps implemented in the image processing software are given in the form of a flow chart in Figure 1.

Thanks to its low computational cost, the developed software operated at approximately real-time speed on a standard laptop computer (Intel i7 processor, 16 GB RAM, Suwon-si, Republic of Korea).

The parameters of the COP and image processing outputs are given in Table 1.

### 2.3. Statistical Analysis

All statistical analyses were performed using IBM SPSS Statistics version 26.0 (SPSS Inc., Chicago, IL, USA). The normality of continuous variables was assessed through visual (histograms, probability plots) and analytical methods (Shapiro–Wilk test, skewness, kurtosis, and coefficient of variation). Descriptive statistics were presented as frequencies and percentages for categorical variables, and as mean ± standard deviation or median (interquartile range) for continuous variables, depending on distribution.

Group comparisons of image processing parameters between children with cerebral palsy (CP) and healthy controls were conducted using the Mann–Whitney U test. Non-normally distributed variables were logarithmically transformed before applying parametric tests. Relationships between continuous variables were analyzed using Pearson correlation, and correlation strength was interpreted based on standard thresholds (very weak to very strong).

To predict force platform parameters from image processing data, simple or multiple linear regression analyses were performed. Variables with correlation coefficients above 0.25 and statistical significance were included in regression models after checking for multicollinearity (threshold > 0.80). All regression models were constructed using the Enter method, and model fit was evaluated using residual and fit statistics. Effect sizes of predictors were interpreted using Cohen’s R^2^ criteria: 0.02 (small), 0.13 (medium), and 0.26 (large). Statistical significance was set at *p* < 0.05.

## 3. Results

A total of 83 children (63 with CP, GMFCS levels I–II; 20 healthy controls) participated in the study. Sociodemographic and clinical characteristics of the participants were analyzed and found to be comparable between the groups, with no significant differences in age, weight, height, or body mass index (*p* > 0.05) (Table 2).

The comparison of image processing parameters between the CP and control groups showed significant differences under all test conditions (*p* < 0.05), confirming impaired postural control in the CP group.

1st Condition: Feet Shoulder-Width Apart, Eyes Open

In the most stable stance condition, image processing parameters demonstrated strong correlations with corresponding force platform parameters. The mean radial velocity (Vmean_COMr1) and total mediolateral displacement (Ctotal_COMx1) were the most predictive variables, with correlation coefficients reaching r = 0.79 and r = 0.76, respectively (*p* < 0.001). Regression analysis showed that these variables explained 74% of the variance (R^2^ = 0.74) in force platform outcomes, indicating very strong predictive capability. The CP group exhibited significantly higher velocity and displacement values compared to controls (*p* < 0.001).

2nd Condition: Feet Shoulder-Width Apart, Eyes Closed

With the removal of visual feedback, the predictive strength slightly decreased. Vmean_COMr2 and total anteroposterior displacement (Ctotal_COMy2) emerged as significant predictors (r = 0.68, r = 0.65, *p* < 0.001), with a regression model explaining 62% of the variance (R^2^ = 0.62). The absence of vision increased postural sway in both groups but was more pronounced in children with CP, confirming the role of visual input in postural control.

3rd Condition: Feet Together, Eyes Open

In this more challenging stance, the predictive power of image processing parameters continued to decline. Standard deviation of radial velocity (Vstd_COMr3) and Ctotal_COMx3 were the most relevant predictors, with moderate correlations to force platform measures (r = 0.59, r = 0.57, *p* < 0.01). The regression model yielded R^2^ = 0.54, indicating a moderate level of explanatory power. The CP group again showed significantly greater variability and displacement (*p* < 0.01), reflecting reduced balance control under narrowed base of support.

4th Condition: Feet Together, Eyes Closed

This condition represented the most unstable posture, combining a narrow stance and lack of visual feedback. The correlations between image processing and force platform data were weaker but still statistically significant (r = 0.51 for Vstd_COMx4, r = 0.49 for Vmean_COMy4, *p* < 0.05). The regression analysis yielded an R^2^ value of 0.42, indicating moderate-to-weak predictive capability. Children with CP showed the largest variability in sway, confirming this condition’s sensitivity to postural instability.

**Figure 2:**
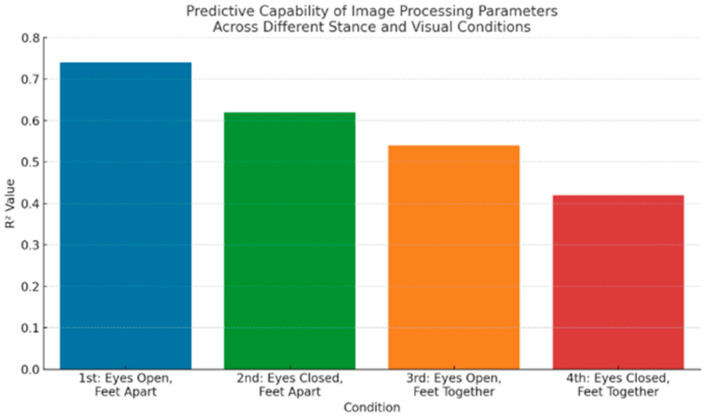
Predictive Capability of Image Processing Parameters Across Different Stance and Visual Conditions; R^2^ Value: predictive power of image processing parameters.

Overall, the image processing parameters showed condition-dependent predictive strength, with the highest accuracy observed under the most stable condition (1st) and progressively decreasing in more challenging scenarios (Figure 2). Mean and standard deviation of velocity, along with total displacement, consistently contributed to the prediction of balance performance across all test conditions.

Detailed correlation and regression results are provided in Supplementary Tables S1.1–S4.3, which present the relationships between COP parameters and image processing data, as well as group comparisons in detail.

## 4. Discussion

This study examined the validity of a newly developed image processing–based software program for assessing static balance in children with cerebral palsy (CP). Traditional objective balance assessment tools, such as force platforms, are considered the gold standard but are limited in routine clinical use due to their high cost and requirement for laboratory settings. The developed software offers an innovative alternative with advantages such as portability, low cost, and ease of application. Importantly, its validation against a force platform adds methodological rigor and enhances the scientific reliability of the findings.

Across four different standing conditions, the balance parameters obtained from the software showed positive correlations—ranging from weak to strong—with center of pressure (COP) data obtained from the force platform. Particularly in the more stable positions (i.e., conditions with eyes open: 1st and 3rd), the predictive power of the software for estimating force platform parameters was statistically significant and moderate. In the most challenging stance (4th condition: feet together and eyes closed), the correlations weakened, suggesting reduced accuracy of the software in complex postural scenarios. Nevertheless, the ability of the system to distinguish between children with CP and typically developing peers under all test conditions supports its diagnostic potential.

Previous literature has consistently reported increased COP and center of mass (COM) sway in individuals with CP, reflecting marked postural instability [24,25,26,27,28]. For instance, Hsue et al. emphasized that mediolateral COM movements could effectively differentiate between hemiplegic, diplegic, and typically developing children. The current study’s findings align with these reports, demonstrating greater sway and increased velocity in all parameters among children with CP. These outcomes were further confirmed by force platform data, reinforcing the validity of the developed software.

There is limited literature on the use of image processing methods for balance assessment specifically in children with CP, which makes this study a pioneering effort. Previous studies in elderly populations or orthopedic patients have suggested that low-cost video analysis may provide meaningful estimates of balance parameters but often suffered from small sample sizes and lack of validation against gold standards [29,30,31]. In contrast, the present study stands out with its adequate sample size, use of a gold-standard reference system, and statistically sound regression models. Notably, the study not only describes postural sway parameters but also provides predictive modeling to quantify the estimation capability of the software.

Although posturography systems such as force platforms and stabilometers are reliable tools for static balance assessment [32,33,34,35], their use is constrained by cost, technical requirements, and lack of portability. Accelerometers, while more portable, still lack standardization and validated protocols for widespread clinical use [36,37]. In this context, the developed image-based system offers several advantages: it requires no physical contact, needs no technical intervention, and operates using only a video recording. These features make it particularly suitable for pediatric use and enhance clinical feasibility.

Deep learning–based pose estimation tools (e.g., OpenPose, DeepLabCut, EfficientPose) can extract joint-level kinematic data with high precision and have been increasingly applied in gait and posture analysis [38,39,40]. These systems have demonstrated strong validity for estimating spatiotemporal gait parameters and postural sway in adults and typically developing populations; however, their clinical translation in children with CP remains limited. One reason is that such tools generally require powerful GPUs, extensive calibration, and controlled recording conditions, which restrict their usability in routine pediatric rehabilitation settings. Moreover, although these methods provide highly detailed kinematic outputs, they often generate large volumes of data that may not be directly applicable or interpretable for clinicians.

The current study adopted a frame-difference–based approach, which prioritizes computational efficiency and clinical feasibility. While it does not provide joint-level precision comparable to OpenPose or DeepLabCut, it focuses on extracting global sway-related parameters that are directly comparable to force platform outcomes. This targeted approach enhances its practicality for balance assessment in children with CP, as it avoids the technical and computational barriers of deep learning–based pose estimation. On the other hand, the available software makes it particularly suitable for low-resource environments, schools, or outpatient clinics where advanced hardware is not available. Moreover, our findings demonstrate that even with this simplified approach, the software was able to capture clinically meaningful sway differences between CP and typically developing children and showed significant correlations with COP parameters from the gold-standard force platform. Thus, while deep learning frameworks remain promising for future iterations, the present solution offers a pragmatic and accessible alternative tailored to clinical practice.

In contrast, the frame difference method implemented in our software was intentionally selected for its computational efficiency and minimal hardware requirements. Unlike deep learning models, which demand high processing power and GPU support, frame differencing can operate effectively on standard clinical computers and even low-end devices, without compromising feasibility in everyday settings. This makes the proposed system particularly suitable for low-resource environments, schools, or outpatient clinics where advanced hardware is not available. While future iterations may incorporate lightweight deep learning architectures (e.g., MobileNetV2, EfficientPose) to further enhance accuracy, the present design prioritizes accessibility, speed, and clinical practicality—attributes that are critical for real-world implementation in pediatric rehabilitation.

Despite these promising findings, several limitations should be acknowledged. First, test–retest reliability of the software was not assessed; validation of intra- and inter-rater reproducibility is essential before clinical adoption. Second, the study included only ambulatory children classified as GMFCS levels I–II; therefore, applicability to individuals with more severe impairments is yet to be determined. Third, the class imbalance in our sample (greater number of children with CP compared to controls) may influence the generalizability of the regression models. Additionally, future studies with larger and more balanced sample groups are needed to increase generalizability. Finally, although software has shown correlations with COP parameters, further comparison with advanced motion capture or wearable sensor systems, or with advanced software that has been proven valid and reliable, may provide stronger evidence of validity and accuracy.

## 5. Conclusions

This study demonstrated that the newly developed image processing–based software provides a valid and clinically feasible alternative for assessing static balance in children with cerebral palsy (CP). By employing a computationally efficient frame-difference method, the system showed significant correlations with center of pressure (COP) measures from a gold-standard force platform and successfully distinguished children with CP from their typically developing peers. Compared with conventional tools such as force platforms, stabilometers, inertial sensors, or deep learning-based pose estimation frameworks, the proposed software offers key advantages of portability, low cost, and ease of use, making it particularly suitable for low-resource environments, schools, and outpatient clinics. As part of a multidisciplinary approach, this technology is expected to contribute to the ongoing digital transformation in physiotherapy and rehabilitation. While the study is limited to ambulatory children (GMFCS I–II) and lacks test–retest reliability data, these findings highlight the software’s potential as a scalable clinical tool. Future research should extend validation to children with more severe impairments, assess reproducibility, and explore the integration of lightweight deep learning architectures to further enhance accuracy while preserving accessibility.

## Figures and Tables

**Table 1:** COP and image processing software parameters.

Parameters	COP	Image Processing Software	Description
Vmean x	Vmean COPx (mm/s)	Vmean COMx (pixel/s)	Average oscillation velocity in the mediolateral direction
Vmean y	Vmean COPy (mm/s)	Vmean COMy (pixel/s)	Average oscillation velocity in the anteroposterior direction
Vmean r	Vmean COPr (mm/s)	Vmean COMr (pixel/s)	Average oscillation velocity
Vstd x	Vstd COPx	Vstd COMx	Standard deviation velocity in the mediolateral direction
Vstd y	Vstd COPy	Vstd COMy	Standard deviation velocity in the anteroposterior direction
Vstd r	Vstd COPr	Vstd COMr	Standard deviation velocity
Ctotal x	Ctotal COPx (mm)	Ctotal COMx (pixel)	Total displacement in the mediolateral direction
Ctotal y	Ctotal COPy (mm)	Ctotal COMy (pixel)	Total displacement in the anteroposterior direction
Ctotal r	Ctotal COPr (mm)	Ctotal COMr (pixel)	Total displacement

x: mediolateral direction, y: anteroposterior direction, r: radial resultant.

**Table 2:** Sociodemographic and Clinical Characteristics of Participants (n = 83).

Sociodemographic and Clinical Characteristics	CP Group (n = 63)	Control Group (n = 20)	*p*
Age (years)	7.95 ± 2.59	8.25 ± 2.53	0.621
Body Weight (kg)	29.54 ± 9.71	32.1 ± 10.42	0.394
Height (cm)	128.13 ± 17.44	132 ± 13.56	0.43
BMI (kg/m^2^)	17.48 ± 2.4	17.92 ± 2.51	0.406
Gender			
Female	23 (36.51%)	11 (55.0%)	0.143
Male	40 (63.49%)	9 (45.0%)	
CP Type			
Unilateral	46 (73.02%)	NA	
Bilateral	17 (26.98%)	NA	
GMFCS			
I	40 (63.49%)	NA	
II	23 (36.51%)	NA	

## Data Availability

Data will be made available upon reasonable request. The data are not publicly available due to [ethical issues that may arise due to the privacy of young children].

## Supplementary Materials

The following supporting information can be downloaded at: https://www.mdpi.com/article/10.3390/healthcare13212682/s1, Table S1.1. Correlation Analysis Between Force Plate and Image Processing Parameters in the Eyes Open, Feet Apart, Arms Alongside the Body Condition. Table S1.2. Regression Model Results Predicting Force Plate Parameters from Image Processing Parameters in the Eyes Open, Feet Apart, Arms Alongside the Body Condition. Table S1.3. Comparison of Balance Parameters Between Children with Cerebral Palsy and the Control Group in the Eyes Open, Feet Apart, Arms Alongside the Body Condition. Table S2.1. Correlation Analysis Between Force Plate and Image Processing Parameters in the Eyes Closed, Feet Apart, Arms Alongside the Body Condition. Table S2.2. Regression Model Results Predicting Force Plate Parameters from Image Processing Parameters in the Eyes Closed, Feet Apart, Arms Alongside the Body Condition. Table S2.3. Comparison of Balance Parameters Between Children with Cerebral Palsy and the Control Group in the Eyes Closed, Feet Apart, Arms Alongside the Body Condition. Table S3.1. Correlation Analysis Between Force Plate and Image Processing Parameters in the Eyes Open, Feet Together, Arms Alongside the Body Condition. Table S3.2. Regression Model Results Predicting Force Plate Parameters from Image Processing Parameters in the Eyes Open, Feet Together, Arms Alongside the Body Condition. Table S3.3. Comparison of Balance Parameters Between Children with Cerebral Palsy and the Control Group in the Eyes Open, Feet Together, Arms Alongside the Body Condition. Table S4.1. Correlation Analysis Between Force Plate and Image Processing Parameters in the Eyes Closed, Feet Together, Arms Alongside the Body Condition. Table S4.2. Regression Model Results Predicting Force Plate Parameters from Image Processing Parameters in the Eyes Closed, Feet Together, Arms Alongside the Body Condition. Table S4.3. Comparison of Balance Parameters Between Children with Cerebral Palsy and the Control Group in the Eyes Closed, Feet Together, Arms Alongside the Body Condition.

## Abbreviations

The following abbreviations are used in this manuscript:

CP	Cerebral Palsy
COP	Center of pressure
COM	Center of mass
GMFCS	Gross Motor Function Classification System
