# Supplementary material for: Evaluation of Static Balance in Children with Cerebral Palsy Using an Innovative Image Processing Software
Source: Healthcare (Basel). 2025 Oct 23;13(21):2682. doi: 10.3390/healthcare13212682 (PMC12607820; doi:10.3390/healthcare13212682)
Supplement: Supplementary file 1 [file healthcare-13-02682-s001.zip › healthcare-3840990-supplementary.pdf]

Condition 1: Eyes open, feet shoulder-width apart

Supplemental Table S1.1. Correlation Analysis Between Force Plate and Image Processing Parameters in the Eyes Open, Feet Apart, Arms Alongside the Body Condition.

		Vmean_COM x1	Vmean_COM y1	Vmean_COM r1	Vstd_COM x1	Vstd_COM y1	Vstd_COM r1
**Vmean_COPx1**	**r**	0.521	0.459	0.510	0.268	0.204	0.163
	**p**	<0.001	<0.001	<0.001	0.034	0.109	0.201
**Vmean_COPy1**	**r**	0.657	0.473	0.582	0.521	0.271	0.351
	**p**	<0.001	<0.001	<0.001	<0.001	0.032	0.005
**Vmean_COPr1**	**r**	0.65	0.502	0.596	0.447	0.258	0.29
	**p**	<0.001	<0.001	<0.001	<0.001	0.041	0.021
		**Vmean_COM x1**	**Vmean_COM y1**	**Vmean_COM r1**	**Vstd_COM x1**	**Vstd_COM y1**	**Vstd_COM r1**
**Vstd_COPx1**	**r**	0.531	0.457	0.513	0.28	0.176	0.142
	**p**	<0.001	<0.001	<0.001	0.026	0.168	0.267
**Vstd_COPy1**	**r**	0.707	0.508	0.629	0.545	0.269	0.345
	**p**	<0.001	<0.001	<0.001	<0.001	0.033	0.006
**Vstd_COPr1**	**r**	0.652	0.506	0.6	0.42	0.199	0.214
	**p**	<0.001	<0.001	<0.001	0.001	0.118	0.092
		**Ctotal_COM x1**	**Ctotal_COM y 1**	**Ctotal_COMr 1**			
**Ctotal_COPx1**	**r**	0.517	0.456	0.507			
	**p**	<0.001	<0.001	<0.001			
**Ctotal_COPy1**	**r**	0.651	0.469	0.577			
	**p**	<0.001	<0.001	<0.001			
**Ctotal_COPr1**	**r**	0.643	0.496	0.590			
	**p**	<0.001	<0.001	<0.001			

Vmean_COP: Average oscillation velocity on the force plate, **Vstd_COP:** Standard deviation velocity on the force plate, **Ctotal_COP**: Total displacement on the force plate, **Vmean_COM:** Average oscillation velocity in the image processing software, **Vstd_COM:** Standard deviation velocity in the image processing software, **Ctotal_COM:** Total displacement in the image processing software, **x:** Mediolateral direction, **y:** Anteroposterior direction, **r:** Radial resultant, **1:** 1. Condition.

Supplemental Table S1.2. Regression Model Results Predicting Force Plate Parameters from Image Processing Parameters in the Eyes Open, Feet Apart, Arms Alongside the Body Condition.

**Dependent variables**		**B (Coefficient)**	**Confidence Interval**	**Beta**	**t**	**p**	**VIF**
**Vmean _COPx 1**	**Sabit**	1.561	1,420/1,703	-	22,096	**<0,001**	-
	**Vmean_COMx1**	0.156	0,096/0,215	0,970	5,203	**<0,001**	3,259
	**Vstd_COMx1**	-0.052	-0,088/-0,016	-0,539	-2,894	**0,005**	3,259
	R^2_{adj}=0.339; Standard error of the prediction: 0.149; The model fit: F = 16.905 ve p<0.001						
**Vmean_COPy1**		**B (Coefficient)**	**Confidence Interval**	**Beta**	**t**	**p**	**VIF**
	**Sabit**	1.405	1,210/1,600	-	14,407	**<0,001**	-
	**Vmean_COMy1**	0.120	0,059/0,182	0,773	3,902	**<0,001**	4,010
	**Vstd_COMx1**	0.044	0,020/0,069	0,406	3,615	**0,001**	1,292
	**Vstd_COMy1**	-0.053	-0,089/-0,017	-0,571	-2,940	**0,005**	3,858
	R^2_{adj}=0.393; Standard error of the prediction: 0.16; The model fit: F = 14,397 ve p<0.001						
**Vmean_COPr1**		**B (Coefficient)**	**Confidence Interval**	**Beta**	**t**	**p**	**VIF**
	**Sabit**	1.702	1,541/1,864	-	21,068	**<0,001**	-
	**Vmean_COMr1**	0.074	0,045/0,103	0,754	5,159	**<0,001**	2,306
	**Vstd_COMr1**	-0.045	-0,074/-0,017	-0,540	-3,181	**0,002**	3,110
	**Vstd_COMx1**	0.035	0,005/0,064	0,359	2,330	**0,023**	2,560
	R^2_{adj}=0.425; Standard error of the prediction: 0.139; The model fit: F =16,302 ve p<0.001						
**Vstd_COPx1**		**B (Coefficient)**	**Confidence Interval**	**Beta**	**t**	**p**	**VIF**
	**Sabit**	1.675	1,507/1,842	-	20,027	**<0,001**	-
	**Vstd_COMx1**	-0.061	-0,103/-0,018	-0,527	-2,845	**0,006**	3,259
	**Vmean_COMx1**	0.185	0,114/0,256	0,970	5,232	**<0,001**	3,259
	R^2_{adj}=0.346; Standard error of the prediction: 0.177; The model fit: F = 17,405 ve p<0.001						
**Vstd_COPy1**		**B (Coefficient)**	**Confidence Interval**	**Beta**	**t**	**p**	**VIF**
	**Sabit**	1.539	1,354/1,724	-	16,622	**<0,001**	-
	**Vstd_COMx1**	0.047	0,024/0,070	0,422	4,050	**<0,001**	1,292
	**Vstd_COMy1**	-0.066	-0,100/-0,032	-0,695	-3,855	**<0,001**	3,858
	**Vmean_COMy1**	0.145	0,086/0,203	0,906	4,932	**<0,001**	4,010
	R^2_{adj}=0.478; Standard error of the prediction: 0.152; The model fit: F =19,933 ve p<0.001						
**Vstd_COPr1**		**B (Coefficient)**	**Confidence Interval**	**Beta**	**t**	**p**	**VIF**
	**Sabit**	1.436	1,260/1,612	-	16,357	**<0,001**	-
	**Vstd_COMx1**	-0.053	-0,098/-0,008	-0,399	-2,362	**0,021**	3,259
	**Vmean_COMx1**	0.216	0,142/0,290	0,984	5,821	**<0,001**	3,259
	R^2_{adj}=0.456; Standard error of the prediction: 0,185; The model fit: F = 26,994 ve p<0.001						
**Ctotal_COPx1**		**B (Coefficient)**	**Confidence Interval**	**Beta**	**t**	**p**	**VIF**
	**Sabit**	2.898	2,815/2,981	-	70,024	**<0,001**	-
	**Ctotal_COMx1**	0.006	0,002/0,010	0,338	2,743	**0,008**	1,396
	**Q_total_1**	0.028	0,007/0,049	0,335	2,717	**0,009**	1,396
	R^2_{adj}=0.325; Standard error of the prediction: 0.151; The model fit: F = 15.952 ve p<0.001						
**Ctotal_COP y1**		**B (Coefficient)**	**Confidence Interval**	**Beta**	**t**	**p**	**VIF**
	**Sabit**	2.883	2,791/2,975	-	62,663	**<0,001**	-
	**Ctotal_COMy1**	0.006	0,002/0,010	0,362	3,006	**0,004**	1,204
	**Q_total_1**	0.024	0,002/0,047	0,258	2,142	**0,036**	1,204
	R^2_{adj}=0.251, Standard error of the prediction: 0.178; The model fit: F =11,388 ve p<0.001						
**Ctotal_COPr1**		**B (Coefficient)**	**Confidence Interval**	**Beta**	**t**	**p**	**VIF**
	**Sabit**	3.062	2,982/3,143	-	76,378	**<0,001**	-
	**Ctotal_COMr1**	0.005	0,002/0,007	0,461	4,000	**<0,001**	1,327
	**Q_total_1**	0.022	0,003/0,041	0,260	2,258	0,028	1,327
	R^2_{adj}=0.379 Standard error of the prediction: 0.144; The model fit: F = 19,950 ve p<0.001						

R^2 Value: predictive power of image processing parameters

Supplemental Table S1.3. Comparison of Balance Parameters Between Children with Cerebral Palsy and the Control Group in the Eyes Open, Feet Apart, Arms Alongside the Body Condition.

Force Plate				Image processing software			
	**CP GROUP (n=63)**	**Control Group (n=20)**	**p**		**CP GROUP (n=63)**	**Control Group (n=20)**	**p**
**Vmean_COPx1**	35.78 (30.16-52.23)	26.85 (22.84-32.19)	**0.001**	**Vmean_COMx1**	2.16 (1.02-2.95)	1.06 (0.4-1.62)	**0.002**
**Vmean_COPy1**	39.47 (26.93-48.46)	25.1 (19.76-33.59)	**0.001**	**Vmean_COMy1**	2.48 (0.99-3.27)	1.17 (0.41-1.53)	**<0.001**
**Vmean_COPr1**	64.14 (46.73-79.28)	41.3 (33.52-51.49)	**<0.001**	**Vmean_COMr1**	3.59 (1.7-5.09)	1.73 (0.6-2.52)	**0.001**
**Vstd_COPx1**	44.54 (39.69-64.08)	33.94 (29.41-40.34)	**0.001**	**Vstd_COMx1**	6.19±1.9	4.86±2.25	**0.011**
**Vstd_COPy1**	53.72 (33.71-76.87)	31.67 (25.21-41.96)	**0.001**	**Vstd_COMy1**	7.41±2.23	5.49±2.52	**0.002**
**Vstd_COPr1**	35.24 (26.43-52.04)	24.07 (19.39-27.91)	**<0.001**	**Vstd_COMr1**	8.99±2.18	7.07±3.03	**0.015**
**Ctotal_COPx1**	1072.57 (904.3-1565.89)	805.09 (684.66-965.17)	**0.001**	**Ctotal_COMx1**	19.9 (9.54-27.55)	9.75 (3.74-15.37)	**0.002**
**Ctotal_COPy1**	1183.36 (807.38-1452.98)	752.61 (592.35-1006.91)	**0.001**	**Ctotal_COMy1**	22.73 (9.3-30.98)	10.79 (3.77-14.26)	**<0.001**
**Ctotal_COPr1**	1922.92 (1400.87-2376.93)	1238.14 (1005.06-1543.64)	**<0.001**	**Ctotal_COMr1**	33.54 (15.98-47.17)	15.91 (5.54-23.93)	**0.001**

Vmean_COP: Average oscillation velocity on the force plate, **Vstd_COP:** Standard deviation velocity on the force plate, **Ctotal_COP**: Total displacement on the force plate, **Vmean_COM:** Average oscillation velocity in the image processing software, **Vstd_COM:** Standard deviation velocity in the image processing software, **Ctotal_COM:** Total displacement in the image processing software, **x:** Mediolateral direction, **y:** Anteroposterior direction, **r:** Radial resultant, **1:** 1. Condition.

Condition 2: Eyes closed, feet shoulder-width apart

Supplemental Table S2.1. Correlation Analysis Between Force Plate and Image Processing Parameters in the Eyes Closed, Feet Apart, Arms Alongside the Body Condition.

		**Vmean_COMx2**	**Vmean_COMy2**	**Vmean_COMr2**	**Vstd_COMx2**	**Vstd_COMy2**	**Vstd_COMr2**
**Vmean_COPx2**	**r**	0.348	0.432	0.418	0.056	0.129	0.016
	**p**	0.005	<0.001	0.001	0.664	0.312	0.901
**Vmean_COPy2**	**r**	0.401	0.465	0.458	0.186	0.204	0.129
	**p**	0.001	<0.001	<0.001	0.145	0.108	0.314
**Vmean_COPr2**	**r**	0.416	0.476	0.475	0.143	0.173	0.081
	**p**	0.001	<0.001	<0.001	0.262	0.174	0.53
		**Vmean_COMx2**	**Vmean_COMy2**	**Vmean_COMr2**	**Vstd_COMx2**	**Vstd_COMy2**	**Vstd_COMr2**
**Vstd_COPx2**	**r**	0.366	0.411	0.418	0.071	0.091	-0.002
	**p**	0.003	0.001	0.001	0.578	0.479	0.989
**Vstd_COPy2**	**r**	0.424	0.485	0.484	0.183	0.183	0.105
	**p**	0.001	<0.001	<0.001	0.152	0.151	0.413
**Vstd_COPr2**	**r**	0.431	0.466	0.482	0.129	0.107	0.023
	**p**	<0.001	<0.001	<0.001	0.315	0.404	0.86
		**Ctotal_COMx2**	**Ctotal_COMy2**	**Ctotal_COMr2**			
**Ctotal_COPx2**	**r**	0.359	0.446	0.432			
	**p**	0.004	<0.001	<0.001			
**Ctotal_COPy2**	**r**	0.416	0.477	0.473			
	**p**	0.001	<0.001	<0.001			
**Ctotal_COPr2**	**r**	0.431	0.491	0.491			
	**p**	<0.001	<0.001	<0.001			

Vmean_COP: Average oscillation velocity on the force plate, **Vstd_COP:** Standard deviation velocity on the force plate, **Ctotal_COP**: Total displacement on the force plate, **Vmean_COM:** Average oscillation velocity in the image processing software, **Vstd_COM:** Standard deviation velocity in the image processing software, **Ctotal_COM:** Total displacement in the image processing software, **x:** Mediolateral direction, **y:** Anteroposterior direction, **r:** Radial resultant, **2:** 2. Condition.

Supplemental Table S2.2. Regression Model Results Predicting Force Plate Parameters from Image Processing Parameters in the Eyes Closed, Feet Apart, Arms Alongside the Body Condition.

Dependent variables		B (Coefficient)	Confidence Interval	Beta	t	p	VIF
**Vmean_COPx2**	**Sabit**	1,490	1,396/1,583	-	31,863	**<0,001**	-
	**Vmean_COMx2**	0,052	0,016/0,088	0,348	2,898	**0,005**	1,000
	R^2=0.121. Standard error of the prediction: 0.175<br>The model fit: ANOVA test; F = 8.397 ve p=0.005						
**Vmean_COPy2**		**B (Coefficient)**	**Confidence Interval**	**Beta**	**t**	**p**	**VIF**
	**Sabit**	1,410	1,301/1,518	-	25,947	**<0,001**	-
	**Vmean_COMy2**	0,077	0,039/0,114	0,465	4,101	**<0,001**	1,000
	R^2=0.216. Standard error of the prediction: 0.192<br>The model fit: ANOVA test; F = 16.820 ve p<0.001						
**Vmean_COPr2**		**B (Coefficient)**	**Confidence Interval**	**Beta**	**t**	**p**	**VIF**
	**Sabit**	1,627	1,529/1,725	-	33,179	**<0,001**	-
	**Vmean_COMr2**	0,048	0,025/0,070	0,475	4,211	**<0,001**	1,000
	R^2=0.225. Standard error of the prediction: 0.168<br>The model fit: ANOVA test; F = 17.731 ve p<0.001						
**Vstd_COPx2**		**B (Coefficient)**	**Confidence Interval**	**Beta**	**t**	**p**	**VIF**
	**Sabit**	1,592	1,491/1,692	-	31,630	**<0,001**	-
	**Vmean_COMx2**	0,060	0,021/0,098	0,366	3,069	**0,003**	1,000
	R^2=0.134.. Standard error of the prediction: 0.18817<br>The model fit: ANOVA test; F = 9.420 ve p=0.003						
**Vstd_COPy2**		**B (Coefficient)**	**Confidence Interval**	**Beta**	**t**	**p**	**VIF**
	**Sabit**	1,516	1,407/1,625	-	27,694	**<0,001**	-
	**Vmean_COMy2**	0,082	0,044/0,119	0,485	4,336	**<0,001**	1,000
	R^2=0.236. Standard error of the prediction: 0.19343<br>The model fit: ANOVA test; F = 18.797 ve p<0.001						
**Vstd_COPr2**		**B (Coefficient)**	**Confidence Interval**	**Beta**	**t**	**p**	**VIF**
	**Sabit**	1,384	1,262/1,505	-	22,845	**<0,001**	-
	**Vmean_COMr2**	0,060	0,032/0,088	0,482	4,300	**<0,001**	1,000
	R^2=0.233. Standard error of the prediction: 0.20751<br>The model fit: ANOVA test; F = 18.492 ve p<0.001						
**Ctotal_COPx2**		**B (Coefficient)**	**Confidence Interval**	**Beta**	**t**	**p**	**VIF**
	**Sabit**	2,958	2,872/3,044	-	69,027	**<0,001**	-
	**Ctotal_COMx2**	0,004	0,001/0,008	0,257	2,284	**0,026**	1,066
	**Q_total_2**	0,032	0,014/0,050	0,408	3,625	**0,001**	1,066
	R^2adj=0.262. Standard error of the prediction: 0.15891<br>The model fit: ANOVA test; F = 11.985 ve p<0.001						
**Ctotal_COPy2**		**B (Coefficient)**	**Confidence Interval**	**Beta**	**t**	**p**	**VIF**
	**Sabit**	2,880	2,771/2,988	-	53,092	**<0,001**	-
	**Ctotal_COMy2**	0,008	0,004/0,012	0,477	4,243	**<0,001**	1,000
	R^2=0.228. Standard error of the prediction: 0.19052<br>The model fit: ANOVA test; F =18.003 ve p<0.001						
**Ctotal_COPr2**		**B (Coefficient)**	**Confidence Interval**	**Beta**	**t**	**p**	**VIF**
	**Sabit**	3,100	3,006/3,195	-	65,610	**<0,001**	-
	**Ctotal_COMr2**	0,005	0,002/0,007	0,415	3,694	**<0,001**	1,090
	**Q_total_2**	0,021	0,003/0,040	0,266	2,366	0,021	1,090
	R^2adj=0.283. Standard error of the prediction: 0.16038<br>The model fit: ANOVA test; F = 13.216 ve p<0.001						

R^2 Value: predictive power of image processing parameters

Supplemental Table S2.3. Comparison of Balance Parameters Between Children with Cerebral Palsy and the Control Group in the Eyes Closed, Feet Apart, Arms Alongside the Body Condition.

**Force Plate**				**Image processing software**			
	**CP GROUP (n=63)**	**Control Group (n=20)**	**p**		**CP GROUP (n=63)**	**Control Group (n=20)**	**p**
**Vmean_COPx2**	36.39 (32.84-57.64)	25.98 (24.14-33.92)	**<0.001**	**Vmean_COMx2**	2.5 (1.13-3.11)	1.04 (0.27-1.63)	**0.001**
**Vmean_COPy2**	39.8 (26.99-59.18)	24.99 (20.72-35.34)	**<0.001**	**Vmean_COMy2**	2.87 (1.62-3.84)	0.9 (0.31-1.51)	**<0.001**
**Vmean_COPr2**	62.63 (46.55-84.5)	41.21 (34.98-52.42)	**<0.001**	**Vmean_COMr2**	4.67 (2.42-5.29)	1.64 (0.44-2.5)	**<0.001**
**Vstd_COPx2**	46.83 (41.47-74.73)	33.73 (30.38-43.11)	**<0.001**	**Vstd_COMx2**	5.44 (4.14-7.68)	4.97 (2.45-7.03)	0.115
**Vstd_COPy2**	54.66 (34.32-79.8)	32.18 (26.45-44.53)	**<0.001**	**Vstd_COMy2**	7.7 (6.05-9.38)	4.85 (3.27-7.03)	**<0.001**
**Vstd_COPr2**	41.64 (27.37-66.81)	23.86 (20.9-29.08)	**<0.001**	**Vstd_COMr2**	8.46 (7.26-11.18)	7.2 (4.15-9.29)	**0.016**
**Ctotal_COPx2**	1091.01 (984.62-1727.93)	778.85 (723.81-1016.94)	**<0.001**	**Ctotal_COMx2**	23.28 (10.6-28.65)	9.55 (2.51-15.33)	**0.002**
**Ctotal_COPy2**	1193.28 (809.06-1774.13)	749.06 (621.04-1059.63)	**<0.001**	**Ctotal_COMy2**	26.56 (14.82-35.37)	8.38 (2.83-14.14)	**<0.001**
**Ctotal_COPr2**	1877.57 (1395.72-2533.39)	1235.56 (1048.71-1571.45)	**<0.001**	**Ctotal_COMr2**	42.7 (22.11-50.01)	15.31 (4.03-23.48)	**<0.001**

Vmean_COP: Average oscillation velocity on the force plate, **Vstd_COP:** Standard deviation velocity on the force plate, **Ctotal_COP**: Total displacement on the force plate, **Vmean_COM:** Average oscillation velocity in the image processing software, **Vstd_COM:** Standard deviation velocity in the image processing software, **Ctotal_COM:** Total displacement in the image processing software, **x:** Mediolateral direction, **y:** Anteroposterior direction, **r:** Radial resultant, **2:** 2. Condition.

Condition 3: Eyes open, feet together

Supplemental Table S3.1. Correlation Analysis Between Force Plate and Image Processing Parameters in the Eyes Open, Feet Together, Arms Alongside the Body Condition.

		Vmean_COMx3	Vmean_COMy3	Vmean_COMr3	Vstd_COMx3	Vstd_COMy3	Vstd_COMr3
**Vmean_COPx3**	**r**	0.411	0.518	0.498	0.129	0.221	0.064
	**p**	0.001	<0.001	<0.001	0.313	0.082	0.62
**Vmean_COPy3**	**r**	0.258	0.401	0.356	-0.028	0.174	-0.035
	**p**	0.041	0.001	0.004	0.829	0.174	0.784
**Vmean_COPr3**	**r**	0.342	0.473	0.439	0.047	0.206	0.013
	**p**	0.006	<0.001	<0.001	0.714	0.106	0.918
		**Vmean_COMx3**	**Vmean_COMy3**	**Vmean_COMr3**	**Vstd_COMx3**	**Vstd_COMy3**	**Vstd_COMr3**
**Vstd_COPx3**	**r**	0.409	0.513	0.495	0.124	0.198	0.041
	**p**	0.001	<0.001	<0.001	0.333	0.12	0.751
**Vstd_COPy3**	**r**	0.283	0.423	0.382	-0.004	0.186	-0.019
	**p**	0.024	0.001	0.002	0.976	0.144	0.881
**Vstd_COPr3**	**r**	0.367	0.489	0.463	0.082	0.206	0.026
	**p**	0.003	<0.001	<0.001	0.523	0.106	0.839
		**Ctotal_COMx3**	**Ctotal_COMy3**	**Ctotal_COMr3**			
**Ctotal_COPx3**	**r**	0.412	0.519	0.499			
	**p**	0.001	<0.001	<0.001			
**Ctotal_COPy3**	**r**	0.245	0.386	0.343			
	**p**	0.053	0.002	0.006			
**Ctotal_COPr3**	**r**	0.335	0.464	0.431			
	**p**	0.007	<0.001	<0.001			

Vmean_COP: Average oscillation velocity on the force plate, **Vstd_COP:** Standard deviation velocity on the force plate, **Ctotal_COP**: Total displacement on the force plate, **Vmean_COM:** Average oscillation velocity in the image processing software, **Vstd_COM:** Standard deviation velocity in the image processing software, **Ctotal_COM:** Total displacement in the image processing software, **x:** Mediolateral direction, **y:** Anteroposterior direction, **r:** Radial resultant, **3:** 3. Condition.

Supplemental Table S3.2. Regression Model Results Predicting Force Plate Parameters from Image Processing Parameters in the Eyes Open, Feet Together, Arms Alongside the Body Condition.

Dependent variables		B (Coefficient)	Confidence Interval	Beta	t	p	VIF
**Vmean_COPx3**	**Sabit**	1,423	1,329/1,516	-	30,473	**<0,001**	-
	**Vmean_COMx3**	0,051	0,022/0,080	0,411	3,520	**0,001**	1,000
	R^2=0.169. Standard error of the prediction: 0.159<br>The model fit: ANOVA test; F = 12.391 ve p=0.001						
**Vmean_COPy3**		**B (Coefficient)**	**Confidence Interval**	**Beta**	**t**	**p**	**VIF**
	**Sabit**	1,426	1,307/1,546	-	23,934	**<0,001**	-
	**Vmean_COMy3**	0,055	0,023/0,088	0,401	3,415	**0,001**	1,000
	R^2=0.160. Standard error of the prediction: 0.182<br>The model fit: ANOVA test; F = 11.662 ve p=0.001						
**Vmean_COPr3**		**B (Coefficient)**	**Confidence Interval**	**Beta**	**t**	**p**	**VIF**
	**Sabit**	1,605	1,499/1,712	-	30,162	**<0,001**	-
	**Vmean_COMr3**	0,037	0,018/0,057	0,439	3,819	**<0,001**	1,000
	R^2=0.193. Standard error of the prediction: 0.161<br>The model fit: ANOVA test; F = 14.587 ve p<0.001						
**Vstd_COPx3**		**B (Coefficient)**	**Confidence Interval**	**Beta**	**t**	**p**	**VIF**
	**Sabit**	1,530	1,434/1,627	-	31,778	**<0,001**	-
	**Vmean_COMx3**	0,052	0,022/0,082	0,409	3,500	**0,001**	1,000
	R^2=0.167. Standard error of the prediction: 0.164<br>The model fit: ANOVA test; F = 12.251 ve p=0.001						
**Vstd_COPy3**		**B (Coefficient)**	**Confidence Interval**	**Beta**	**t**	**p**	**VIF**
	**Sabit**	1,523	1,406 1,640	-	26,071	**<0,001**	-
	**Vmean_COMy3**	0,058	0,026/0,090	0,423	3,644	**0,001**	1,000
	R^2=0.179. Standard error of the prediction: 0.179<br>The model fit: ANOVA test; F = 13.276 ve p=0.001						
**Vstd_COPr3**		**B (Coefficient)**	**Confidence Interval**	**Beta**	**t**	**p**	**VIF**
	**Sabit**	1,349	1,234/1,463	-	23,561	**<0,001**	-
	**Vmean_COMr3**	0,043	0,022/0,064	0,463	4,075	**<0,001**	1,000
	R^2=0.214. Standard error of the prediction: 0.173<br>The model fit: ANOVA test; F = 16.610 ve p<0.001						
**Ctotal_COPx3**		**B (Coefficient)**	**Confidence Interval**	**Beta**	**t**	**p**	**VIF**
	**Sabit**	2,898	2,803/2,992	-	61,515	**<0,001**	-
	**Ctotal_COMx3**	0,006	0,002/0,009	0,412	3,529	**0,001**	1,000
	R^2_{adj}=0.170. Standard error of the prediction: 0.159<br>The model fit: ANOVA test; F = 12.454 ve p=0.001						
**Ctotal_COPy3**		**B (Coefficient)**	**Confidence Interval**	**Beta**	**t**	**p**	**VIF**
	**Sabit**	2,909	2,789/3,030	-	48,313	**<0,001**	-
	**Ctotal_COMy3**	0,006	0,002/0,009	0,386	3,272	**0,002**	1,000
	R^2=0.149. Standard error of the prediction: 0.183<br>The model fit: ANOVA test; F =10.703 ve p=0.002						
**Ctotal_COPr3**		**B (Coefficient)**	**Confidence Interval**	**Beta**	**t**	**p**	**VIF**
	**Sabit**	3,084	2,976/3,192	-	57,252	**<0,001**	-
	**Ctotal_COMr3**	0,004	0,002/0,006	0,431	3,734	**<0,001**	1,000
	R^2_{adj}=0.186. Standard error of the prediction: 0.161<br>The model fit: ANOVA test; F = 13.939 ve p<0.001						

R^2 Value: predictive power of image processing parameters

Supplemental Table S3.3. Comparison of Balance Parameters Between Children with Cerebral Palsy and the Control Group in the Eyes Open, Feet Together, Arms Alongside the Body Condition.

**Force Plate**				**Image processing software**			
	**CP GROUP (n=63)**	**Control Group (n=20)**	**p**		**CP GROUP (n=63)**	**Control Group (n=20)**	**p**
**Vmean_COPx3**	40.44 (24.84-48.35)	25.24 (20.72-34.76)	**0.003**	**Vmean_COMx3**	2.72 (1.72-3.66)	1.46 (0.52-2.61)	**0.001**
**Vmean_COPy3**	39.44 (29.28-56.12)	22.72 (18.5-33.77)	**<0.001**	**Vmean_COMy3**	3.38 (2.37-4.27)	1.27 (0.54-2.48)	**<0.001**
**Vmean_COPr3**	70.85 (45.84-79.99)	39.59 (31.06-51.95)	**<0.001**	**Vmean_COMr3**	4.72 (3.53-6.33)	2.11 (0.86-4.24)	**<0.001**
**Vstd_COPx3**	50.67 (31.42-66.23)	31.66 (26.29-43.65)	**0.002**	**Vstd_COMx3**	6.76 (5.17-7.87)	6.13 (3.75-7.12)	**0.046**
**Vstd_COPy3**	50,35 (39,4-72,42)	28,9 (23,69-42,84)	**<0,001**	**Vstd_COMy3**	8,4 (7,45-9,89)	6,01 (4,23-8)	**0,001**
**Vstd_COPr3**	39,41 (28,01-53,73)	21,23 (17,47-30,06)	**<0,001**	**Vstd_COMr3**	9,63 (8,77-10,77)	7,89 (5,88-10,16)	**0,014**
**Ctotal_COPx3**	1212,5 (744,78-1449,57)	756,75 (621,08-1042,19)	**0,003**	**Ctotal_COMx3**	26,59 (16,18-34,46)	13,53 (4,85-23,98)	**<0,001**
**Ctotal_COPy3**	1182,36 (877,71-1682,49)	681,17 (554,52-1012,46)	**<0,001**	**Ctotal_COMy3**	32,11 (23,02-39,86)	11,63 (5,12-22,76)	**<0,001**
**Ctotal_COPr3**	2124,07 (1374,41-2398,04)	1186,83 (931,07-1557,4)	**<0,001**	**Ctotal_COMr3**	46,09 (34,07-58,68)	19,49 (7,97-38,93)	**<0,001**

Vmean_COP: Average oscillation velocity on the force plate, **Vstd_COP:** Standard deviation velocity on the force plate, **Ctotal_COP**: Total displacement on the force plate, **Vmean_COM:** Average oscillation velocity in the image processing software, **Vstd_COM:** Standard deviation velocity in the image processing software, **Ctotal_COM:** Total displacement in the image processing software, **x:** Mediolateral direction, **y:** Anteroposterior direction, **r:** Radial resultant, **3:** 3. Condition.

Condition 4: Eyes closed, feet together

Supplemental Table S4.1. Correlation Analysis Between Force Plate and Image Processing Parameters in the Eyes Closed, Feet Together, Arms Alongside the Body Condition.

		Vmean_COMx4	Vmean_COMy4	Vmean_COMr4	Vstd_COMx4	Vstd_COMy4	Vstd_COMr4
**Vmean_COPx4**	**r**	0.074	0.295	0.212	-0.231	0.062	-0.21
	**p**	0.567	0.019	0.095	0.069	0.627	0.098
**Vmean_COPy4**	**r**	0.174	0.362	0.295	-0.056	0.177	-0.027
	**p**	0.172	0.004	0.019	0.662	0.166	0.834
**Vmean_COPr4**	**r**	0.132	0.35	0.27	-0.15	0.127	-0.125
	**p**	0.301	0.005	0.032	0.24	0.321	0.33
		**Vmean_COMx4**	**Vmean_COMy4**	**Vmean_COMr4**	**Vstd_COMx4**	**Vstd_COMy4**	**Vstd_COMr4**
**Vstd_COPx4**	**r**	0.062	0.252	0.182	-0.232	0.009	-0.242
	**p**	0.627	0.046	0.154	0.067	0.946	0.056
**Vstd_COPy4**	**r**	0.167	0.346	0.283	-0.07	0.146	-0.055
	**p**	0.19	0.005	0.025	0.587	0.254	0.668
**Vstd_COPr4**	**r**	0.099	0.254	0.198	-0.162	0.013	-0.193
	**p**	0.441	0.044	0.12	0.205	0.92	0.129
		**Ctotal_COMx4**	**Ctotal_COMy4**	**Ctotal_COMr4**			
**Ctotal_COPx4**	**r**	0.071	0.296	0.211			
	**p**	0.58	0.019	0.097			
**Ctotal_COPy4**	**r**	0.175	0.362	0.295			
	**p**	0.17	0.004	0.019			
**Ctotal_COPr4**	**r**	0.131	0.35	0.269			
	**p**	0.305	0.005	0.033			

Vmean_COP: Average oscillation velocity on the force plate, **Vstd_COP:** Standard deviation velocity on the force plate, **Ctotal_COP**: Total displacement on the force plate, **Vmean_COM:** Average oscillation velocity in the image processing software, **Vstd_COM:** Standard deviation velocity in the image processing software, **Ctotal_COM:** Total displacement in the image processing software, **x:** Mediolateral direction, **y:** Anteroposterior direction, **r:** Radial resultant, **4:** 4. Condition.

Supplemental Table S4.2. Regression Model Results Predicting Force Plate Parameters from Image Processing Parameters in the Eyes Closed, Feet Together, Arms Alongside the Body Condition.

Dependent variables		**B (Coefficient)**	**Confidence Interval**	**Beta**	**t**	**p**	**VIF**
**Vmean _COPx _4**	**Sabit**	1.523	1.428/1.619	-	31.875	**<0.001**	-
	**Vmean_COMy4**	0.027	0.005/0.049	0.295	2.416	**0.019**	1.000
	R^2=0.087. Standard error of the prediction: 0.152<br>The model fit: ANOVA test; F = 5.835 ve p=0.019						
**Vmean_COP y4**		**B (Coefficient)**	**Confidence Interval**	**Beta**	**t**	**p**	**VIF**
	**Sabit**	1.503	1.407/1.599	-	31.309	**<0.001**	-
	**Vmean_COMy4**	0.034	0.012/0.056	0.362	3.032	**0.004**	1.000
	R^2=0.131. Standard error of the prediction: 0.153<br>The model fit: ANOVA test; F = 9.192 ve p=0.004						
**Vmean_COP r4**		**B (Coefficient)**	**Confidence Interval**	**Beta**	**t**	**p**	**VIF**
	**Sabit**	1.735	1.640/1.831	-	36.319	**<0.001**	-
	**Vmean_COMr4**	0.016	0.001/0.031	0.270	2.189	**0.032**	1.000
	R^2=0.073. Standard error of the prediction: 0.146<br>The model fit: ANOVA test; F = 4.792 ve p=0.032						
**Ctotal_COPx4**		**B (Coefficient)**	**Confidence Interval**	**Beta**	**t**	**p**	**VIF**
	**Sabit**	2.956	2.870/3.042	-	68.454	**<0.001**	-
	**Ctotal_COMy4**	0.003	0.001/0.005	0.270	2.522	**0.014**	1.003
	**Q_total_4**	0.037	0.021/0.054	0.475	4.433	**<0.001**	1.003
	R^2_{adj}=0.290 Standard error of the prediction: 0.133<br>The model fit: ANOVA test; F = 13.637 ve p<0.001						
**Ctotal_COPy4**		**B (Coefficient)**	**Confidence Interval**	**Beta**	**t**	**p**	**VIF**
	**Sabit**	2.979	2.882/3.075	-	61.682	**<0.001**	-
	**Ctotal_COMy4**	0.004	0.001/0.006	0.362	3.031	**0.004**	1.000
	R^2=0.131. Standard error of the prediction: 0.153<br>The model fit: ANOVA test; F =9.188 ve p=0.004						
**Ctotal_COPr4**		**B (Coefficient)**	**Confidence Interval**	**Beta**	**t**	**p**	**VIF**
	**Sabit**	3.180	3.088/3.272	-	69.050	**<0.001**	-
	**Ctotal_COMr4**	0.002	0.000/0.003	0.251	2.175	**0.034**	1.003
	**Q_total_4**	0.027	0.010/0.045	0.363	3.151	**0.003**	1.003
	R^2_{adj}=0.178. Standard error of the prediction: 0.136<br>The model fit: ANOVA test; F =7.692 ve p=0.001						

R^2 Value: predictive power of image processing parameters

Supplemental Table S4.3. Comparison of Balance Parameters Between Children with Cerebral Palsy and the Control Group in the Eyes Closed, Feet Together, Arms Alongside the Body Condition.

**Force Plate**				**Image processing software**			
	**CP GROUP (n=63)**	**Control Group (n=20)**	**p**		**CP GROUP (n=63)**	**Control Group (n=20)**	**p**
**Vmean_COPx4**	40.8 (32.39-54.62)	25.83 (22.55-35.63)	**<0.001**	**Vmean_COMx4**	3.57 (2.48-4.67)	1.19 (0.61-3.09)	**<0.001**
**Vmean_COPy4**	45.13 (33.43-51.28)	27.57 (21.66-30.21)	**<0.001**	**Vmean_COMy4**	3.89 (2.73-4.66)	0.89 (0.62-2.71)	**<0.001**
**Vmean_COPr4**	69.51 (52.83-83.37)	41.98 (35.8-51.05)	**<0.001**	**Vmean_COMr4**	5.84 (4.05-7.46)	1.65 (1.02-4.49)	**<0.001**
**Ctotal_COPx4**	1223.17 (971.14-1637.48)	774.48 (676.18-1068.1)	**<0.001**	**Ctotal_COMx4**	32.9 (23.53-45.34)	11.16 (5.67-28.39)	**<0.001**
**Ctotal_COPy4**	1353.1 (1002.16-1537.34)	826.42 (649.36-905.67)	**<0.001**	**Ctotal_COMy4**	36.02 (25.64-44.27)	8.19 (5.78-24.93)	**<0.001**
**Ctotal_COPr4**	2083.97 (1583.94-2499.37)	1258.62 (1073.4-1530.43)	**<0.001**	**Ctotal_COMr4**	54.16 (40.36-71.1)	15.24 (9.44-41.44)	**<0.001**

Vmean_COP: Average oscillation velocity on the force plate, **Vstd_COP:** Standard deviation velocity on the force plate, **Ctotal_COP**: Total displacement on the force plate, **Vmean_COM:** Average oscillation velocity in the image processing software, **Vstd_COM:** Standard deviation velocity in the image processing software, **Ctotal_COM:** Total displacement in the image processing software, **x:** Mediolateral direction, **y:** Anteroposterior direction, **r:** Radial resultant, **4:** 4. Condition.
